# Epidemiological, clinical and oncological outcomes of laryngeal verrucous carcinomas: a systematic review

**DOI:** 10.1186/s40463-023-00666-1

**Published:** 2023-12-13

**Authors:** Jérôme R. Lechien, Stéphane Hans

**Affiliations:** 1 Laryngeal and Head and Neck Surgery Study Groups of Young-Otolaryngologists of the International Federations of Oto-rhino-laryngological Societies (YO-IFOS), Paris, France; 2 Department of Otolaryngology, Elsan Hospital, Paris, France; 3 Department of Otolaryngology-Head and Neck Surgery, Foch Hospital, School of Medicine, UFR Simone Veil, Université Versailles Saint-Quentin-en-Yvelines (Paris Saclay University), Paris, France; 4 Division of Laryngology, Department of Otolaryngology-Head and Neck Surgery, Human Anatomy and Experimental Oncology, Faculty of Medicine, UMONS Research Institute for Health Sciences and Technology, University of Mons (UMons), Mons, Belgium; 5 Department of Otorhinolaryngology and Head and Neck Surgery, Foch Hospital, School of Medicine, UFR Simone Veil, Université Versailles Saint-Quentin-en-Yvelines (Paris Saclay University), Paris, France

**Keywords:** Laryngeal, Larynx, Cancer, Squamous cell carcinoma, Otolaryngology, Head neck surgery, Verrucous, Ackerman, Tumor, Voice

## Abstract

**Objective:**

To investigate epidemiological, clinical and oncological outcomes of patients with laryngeal verrucous carcinomas (LVC).

**Methods:**

Two independent authors investigated PubMed, Scopus and Cochrane Library for studies dedicated to epidemiological, clinical and oncological outcomes of patients with LVC. The following outcomes were investigated with PRISMA criteria: age; gender; tobacco/alcohol consumption; HPV infection; anatomical, pathological, therapeutic and survival outcomes. Studies were analyzed for bias through a validated clinical tool.

**Results:**

Of the 212 identified articles, 15 retrospective studies and one prospective uncontrolled study met our inclusion criteria. Three studies reported findings from national databases. The males/females ratio is 9/1. Mean age was 60.3 years, which was younger compared to other laryngeal malignancies. The alcohol, cigarette overuse and the HPV status of patients were lacking in most studies. Glottis and supraglottis were the most common anatomical locations, corresponding to 78.7% and 12.4% of cases, respectively. The main therapeutic approaches consisted of surgery, radiotherapy, surgery followed by radiotherapy. Treatments reported 5-year overall survival and disease-specific survival of 86.3 and 90.8, respectively. The 5- and 10-year local control rate were 83.6 and 72.6, respectively. The 10-year disease-specific survival was 80.2. Heterogeneity between studies was found for inclusion criteria, comorbidity data, and treatments.

**Conclusion:**

LVC is a rare laryngeal cancer associated with better survival and recurrence outcomes than laryngeal squamous cell carcinoma. The role of radiotherapy in the treatment regimen needs to be investigated in future prospective controlled studies**.**

## Introduction

Head and neck squamous cell carcinomas are the 6th most common adult malignancy worldwide, corresponding to 5.3% of all cancers [1]. Laryngeal cancer (LC) is the second most common head and neck cancer, accounting for 211,000 new cases and 126,000 deaths yearly worldwide, respectively [1]. Laryngeal verrucous carcinoma (LVC) is a well-differentiated variant of laryngeal squamous cell carcinoma (LSCC) and accounts for 1–3.4% of laryngeal malignancies [2, 3]. Since the first case reported by Ackerman in 1948 [4], the management strategies of LVC remain controversial. Surgery has long time been considered as the treatment of choice with overall survival and disease-free survival rates ranging from 86.8 to 100% [2, 3, 5]. The overall survival (OS) and disease-free survival (DFS) of radiotherapy were both lower than surgery with up to 70% of patients with disease free at follow-up [2, 3, 6, 7]. The radiotherapy was moreover controversial regarding the potential risk of anaplastic transformation after treatment [8]. The discussions about the most effective treatment continue regarding recent studies supporting the usefulness of radiation approaches for the treatment of LVC [6, 7].

The aim of this systematic review was to investigate epidemiological, clinical and oncological outcomes of patients with laryngeal verrucous carcinomas (LVC).

## Material and methods

The criteria for study inclusion were based on the population, intervention, comparison, outcome, timing and setting (PICOTS) framework [9]. Two authors (JRL and SH) independently reviewed and extracted data according to the PRISMA checklist for systematic reviews [10].

### Eligibility criteria

Prospective and retrospective, controlled, uncontrolled, or randomized studies published between January 1960 and Mai 2023 were included if they investigated epidemiological, pathological, therapeutic or oncological outcomes of LVC patients. Authors had to describe the method of diagnostic of LVC, including pathological features or use of international classifications. To date, LVC is considered as a low-grade variant of LSCC with specific morphologic, cytokinetic and clinical features. Microscopically, verrucous carcinoma consists of filiform projections lined by thick, well-differentiated keratinized squamous epithelium, composed of one to a few layers of basal cells, and multiplied, voluminous spinous cells lacking cytological atypia. This carcinoma may invade the stroma with a well-defined, pushing margin [11]. The studies had to be published in English, Spanish, or French peer-reviewed journals. Authors only considered studies reporting data for more than 10 individuals.

### Populations, inclusion/exclusion criteria

Authors had to describe inclusion criteria of patients, and, particularly, the pathological features considered for the LVC diagnostic. Controlled studies comparing patients with LVC versus individuals with LSCC were considered. The authors used the levels of evidence (I-V) to characterize the studies [12].

### Outcomes

Two authors (JRL and SH) reviewed the following outcomes: number of patients; criteria of LVC diagnostic; gender ratio; tobacco/alcohol overuse; history of human papilloma virus (HPV) infection; mean or median age; LVC location (glottis, supra- or subglottis, transglottis); tumor stages; therapeutic approaches; and oncological outcomes.

The Tool to Assess Risk of Bias in Cohort Studies developed by the Clarity Group and Evidence Partners was used by two authors (JRL and SH) for the bias/heterogeneity analyses of the included studies [13]. The bias analysis included the evaluation of cofactors that may influence the studies findings, i.e. epidemiological (comorbidities, tobacco/alcohol use, etc.), clinical, histopathological and therapeutic characteristics (radiotherapy protocol).

Authors extracted the following oncological outcomes from studies: OS, DFS, disease-specific survival (DSS), recurrence and second malignancy rates.

### Intervention and comparison

The following therapeutic approaches were reviewed for each study: surgery; radiotherapy; chemotherapy; combined treatments; or lack of treatment. The period of the inclusion of patients was considered in the therapeutic analysis regarding the evolution of some therapeutic procedures over the past decades (i.e. chemotherapy, radiotherapy).

### Timing and setting

There was no criteria for specific stage or timing in the ‘disease process’ of the study population. Data from population-based registries or clinical hospital studies were considered.

### Search strategy

The paper search was carried out by three independent authors (JRL and SH) with PubMED, Scopus, and Cochrane Library databases. The databases were screened for abstracts and titles referring to the description of features of LVC patients. Authors analyzed full texts of the selected papers. Results of the search strategy were reviewed for relevance and the reference lists were examined for additional pertinent studies. Any discrepancies in synthesized data were discussed and resolved by the remaining co-authors. The following MeSH/keywords were considered: ‘larynx’; ‘laryngeal’; ‘cancer’; ‘verrucous’; ‘carcinoma’; ‘treatment’; ‘survival’; ‘outcomes’.

## Results

**Fig. 1:**
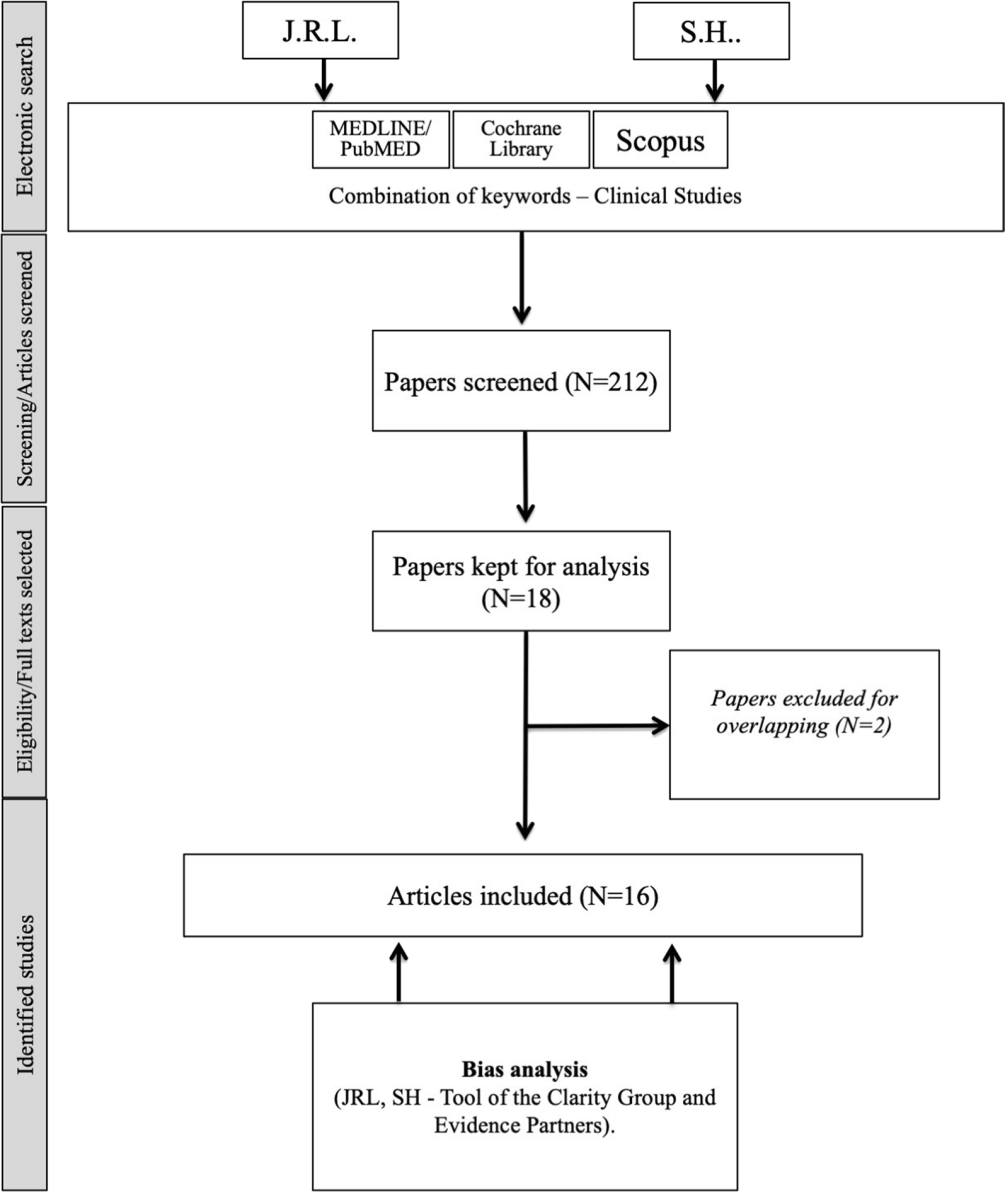
Chart flow

A total of 212 articles were identified. Among them, 16 studies published between 1976 and 2020 met our inclusion criteria (Fig. 1) [3, 8, 12, 14–26]. Two studies were excluded because overlapping with other case-series [27, 28]. Fifteen studies were retrospective (EL: IV) and there was a prospective uncontrolled study (EL: III) (Table 1). Three studies reported findings from national databases, i.e. the Slovenian national database [22], national cancer database [25], and surveillance, epidemiology, and end results (SEER) [3]. There would be potential overlaps between the two U.S. database studies [3, 25] but authors did not investigate similar findings. The present systematic review reported the features of 948 patients with LVC (Table 2). The characteristics of Table 2 do not include the data of one study [25] because risk of overlapping and, moreover, authors focused on specific population of LVC patients that did not match with the aims of the review. Thus, the patient data of the study of Dubal et al. [3] were only considered, while the data of Jayakrishnan et al. [25] were not included in the data of Table 2.

**Table 1:** Included studies

References	Countries	Center	Year	Design	EL	N	Treatments
Burns et al. [12]	Canada	Toronto	1976	Retrospective	IV	18	Surgery and RT
Ferlito and Recher [26]	Italy	Padua	1980	Retrospective	IV	77	Surgery and RT
Blakeslee et al. [14]	U.S.A	Boston	1984	Prospective	III	12	Surgery
Lundgren et al. [8]	Canada	Toronto	1986	Retrospective	IV	44	Surgery and RT
Sllamniky et al. [15]	U.S.A	St Louis	1989	Retrospective	IV	15	Surgery
Hagen et al. [16]	U.S.A	New Orleans	1993	Retrospective	IV	12	Surgery and RT
Fliss et al. [17]	Canada	Toronto	1994	Retrospective	IV	29	Surgery and RT
Longarela Herrero et al. [18]	Spain	Santader	1995	Retrospective	IV	11	Surgery
Maurizi et al. [19]	Italy	Rome	1996	Retrospective	IV	31	Surgery and RT
Damm et al. [20]	Germany	Cologne	1997	Retrospective	IV	21	Surgery
McCaffrey et al. [21]	U.S.A	Rochester	1998	Retrospective	IV	52	Surgery and RT
Strojan et al. [22]	Slovenia	National database	2006	Retrospective	IV	30	Surgery, RT and CT
Hod et al. [23]	Israel	Tel Aviv	2009	Retrospective	IV	18	Surgery and RT
Huang et al. [24]	Canada	Toronto	2009	Retrospective	IV	62	RT
Dubal et al. [3]	U.S.A	SEER	2015	Retrospective	IV	516	Surgery and RT
Jayakrishnan et al. [25]	U.S.A	NCDB	2020	Retrospective	IV	396	Surgery and RT

*CR* chemotherapy, *NCDB* National Cancer Database, *RT* radiotherapy, *SEER* surveillance, epidemiology, and end results

**Table 2:** Pooled features of included studies

Characteristics	N tot	N (%)
Patients		948
Gender		
Females	948	95 (10.0)
Males		841 (88.8)
Unspecified		12 (1.2)
Age	911	
Mean (years)		60.3
Median (years)		60.8
Risk factors		
Tobacco	144	111 (77.1)
Alcohol	18	2 (11.1)
Anatomical location		
Glottis	952	749 (78.7)
Supraglottis		118 (12.4)
Subglottis		8 (0.8)
Transglottis		38 (4.0)
Ary-epiglottic fold		2 (0.2)
Unspecified		37 (3.9)
cTNM staging		
cT1	532	223 (41.9)
cT2		155 (29.1)
cT3		140 (26.3)
cT4		14 (2.7)
N+	169	3 (1.8)
M+	169	0 (0)
Stages		
I	281	141 (50.2)
II		91 (32.4)
III		39 (13.9)
IV		10 (3.5)

*N* number, *N*+ nodes+, *M*+ metastasis+

### Epidemiological features

LVC occur in males and females in 88.8% and 10.0% of cases, respectively. The gender proportion was not reported in one study, accounting for 1.2% of cases [14]. Mean or median age were available in 911 cases and were 60.3 and 60.8 years, respectively. Few studies reported tobacco or alcohol overuse [16, 19, 20, 23, 24]. HPV infection was investigated in the study of Fliss et al. who detected HPV-16 and -18 DNA in 13 cases (45%) [17]. Ethnicity data were considered in one SEER study [5]. Among 2039 head and neck verrucous carcinomas, white and black patients accounted for 1765 (86.6%) and 120 (5.9%), respectively. The proportion of black patients was significantly higher in group with head and neck SCC patients compared to head and neck verrucous carcinoma patient group [5]. However, authors did not investigate specific data for LVC. In the study of Dubal et al. the ethnicity analysis reported a white preponderance in LVC (88.4%) and other laryngeal malignancies (82.6%), with blacks constituting 8.1% of LVC and 13.8% of other laryngeal cancers [3].

### Anatomical and pathological features

The tumor location was available in 15 studies [3, 8, 12, 14–24, 26]. Glottis and supraglottis were the most common anatomical locations of LVC, corresponding to 78.7% and 12.4% of cases, respectively (Table 2). In 4.0% of cases, the tumor was transglottic at the time of diagnosis. Compared to other laryngeal malignancies, LVC developed more frequently in glottis and less frequently in supraglottis [3]. The cTNM staging was reported in 14 studies [3, 8, 12, 14–17, 19–25]. Some authors only investigated outcomes of cT1 and/or cT2 LVC [12, 14, 23], excluding data from cT3 and cT4 tumors. Considering cohorts without cT stage restriction, LVC was detected in cT1, cT2, and cT3 stages in 41.9%, 29.1%, and 26.3%, respectively (Table 2). cT4 LVC concerned 2.7% of patients at the diagnostic time. Limited data were available for cN and metastasis staging in the studies of the literature. Patients presented with positive nodes at the pathological examination in 1.8% of cases. In the present systematic review, there was no patient with distant metastasis. Most patients were diagnosed with stage 1 LVC (Table 2).

### Therapeutic strategies

From retrospective studies published between 1976 and 2020, the main therapeutic approaches consisted of surgery, radiotherapy, surgery followed by radiotherapy or chemotherapy (Table 3). Various surgical approaches were reported, including type I–IV cordectomy, partial or total laryngectomy. At the exception of the study of Huang et al. [24] most authors favored surgery for the treatment. Anaplastic transformation of LVC was investigated in 5 studies and reported in 3 cases [8, 12, 16, 21, 24], accounting for 2.1% of cases (3/140). The three reported cases occurred in the seventies and eighties. The radiotherapy effectiveness was reported in two studies [16, 24]. Hagen et al. included data from 12 LVC patients. Among them, 2 were treated with radiotherapy, which failed in one case (50%) [16]. In the study of Huang et al., 62 patients were treated with radiotherapy and 18 individuals (29%) needed salvage surgery because non-response to radiation [24]. Note that most patients of the study of Huang et al. were treated in the seventies, eighties, and nineties. Radiotherapy was most frequently performed in patients with private insurance compared to those without insurance [25].

**Table 3:** Oncological outcomes

Reference	N	Stades	Survival outcomes	Treatment	Rates
Burns et al. [12]	18	cT1 (18)	3-years DFS	Surgery (8)	89.0
				Radiotherapy (8)	85.3
Ferlito and Recher [26]	77	cT1-T4	5-years metastasis	Surgery and RT (6)	0
			5-years local control rate	Surgery (60)	96.7
				Radiotherapy (1)	0
				Surgery + RT (6)	66.4
Blakeslee et al. [14]	12	cT1 (12)	3-years DSS	Surgery (12)	100
			3-years Recurrence		0
Lundgren et al. [8]	44	cT1 (21)	82.2-months RFS	Surgery (16)	75.0
		cT2 (18)		Radiotherapy (28)	43.0
		cT3 (3)			
		cT4 (2)			
Sllamniku et al. [15]	15	cT1 (7)	5-years OS	Surgery (15)	62.5
		cT2 (3)			
		cT3 (5)			
Hagen et al. [16]	12	cT1 (6)	8-years RFS	Surgery (10)	80.0
		cT2 (3)		Radiotherapy (2)	100
		cT3 (4)	6-years DFS	Surgery and RT	83.0
			6-years metastasis rate	Radiotherapy (2)	50
				Surgery (10)	0
Fliss et al. [17]	29	cT1 (12)	16-months OS	Surgery (6)	83.3
		cT2 (9)	62-months OS	Radiotherapy (16)	62.5
		cT3 (1)	40-months recurrence	Surgery (6)	0
		Unknown (7)		Radiotherapy (16)	37.0
Longarela Herrero et al. [18]	11	cT1-T2	5-years OS	Surgery (11)	100
Huang et al. [24]	62	cT1 (19)	5-years local control rate	Radiotherapy (62)	66.0
		cT2 (36)	10-years local control rate		64.0
		cT3 (6)	5-years OS		87.0
		cT4 (1)	10-years OS		64.0
			5-years DSS		97.0
			10-years DSS		95.0
Maurizi et al. [19]	31	cT1 (15)	79-months recurrence	Surgery and RT (31)	23
		cT2 (10)	79-months OS	Surgery and RT (31)	93.3
		cT3 (6)		Surgery (23)	100
				Surgery + RT (7)	71.4
Damm et al. [20]	21	cT1 (14)	52-months DFS	Surgery (21)	85.7
		cT2 (7)	52-months recurrence		5
McCaffrey et al. [21]	52	cT1 (33)	5-years OS	Surgery and RT (52)	96.1
		cT2 (5)	5-years RFS		71.0
		cT3 (12)			
		cT4 (2)			
Strojan et al. [22]	30	cT1 (4)	5-years OS	Surgery, RT and CRT (30)	75.0
		cT2 (11)	5-years DSS		100
		cT3 (12)	5-years LFFS		97.0
		cT4 (3)			
Hod et al. [23]	18	cT1 (18)	48-months recurrence rate	Surgery (13)	7.7
				Surgery + RT (5)	80.0
Dubal et al. [3]	452	cT1 (92)	1-year DFS and RS	Surgery and RT (452)	97.5—98.1
		cT2 (53)	5-years DFS and RS	Surgery and RT (392)	88.0—85.5
		cT3 (21)	10-years DFS and RS	Surgery and RT (339)	77.4—74.2
		cT4 (6)	5-years DSS	Surgery (292)	90.2
				Radiotherapy (48)	80.6
Jayakrishnan et al. [25]	396	cT1 (217)	5-years OS	Surgery (286)	79.0
		cT2 (186)		Radiotherapy (110)	67.0

*DFS* disease-free survival, *DSS* disease-specific survival, *LFFS* local failure free survival, *OS* overall survival, *RFS* recurrent free survival, *RS* relative survival, *RT* radiotherapy

### Oncological outcomes

Oncological outcomes were reported in 15 studies (Table 3) [3, 8, 12, 14–25]. Considering all treatments, the 5-year OS and DSS were 86.3% and 90.8%, respectively. The 5- and 10-year local control rate were 83.6% and 72.6%, respectively. The 10-year DSS was 80.2. Oncological data were analyzed in 13 studies according to the type of treatment [3, 8, 12, 14–20, 23–25]. As found in Table 3, the 5-year OS for surgery and radiotherapy ranged from 62.5 to 100% and 0 to 87.0%, respectively. Because authors assessed OS, DSS and RFS rates at several follow-up time, it is complicated to provide additional rates for treatment-specific oncological outcomes. Moreover, the data of Jayakrishnan et al. were not considered in the assessment of OS, DSS and RFS rates because authors only focused on cTis-T2 LVC [25]. For this specific group of LVC, authors reported 5-years OS for surgery and radiotherapy of 79.0% and 67.0%, respectively. Echanique et al. combined LVC case reports and case-series (N = 282) and reported local recurrence in 33 cases (11.7%) and regional metastasis in 2 cases (0.8%) [2]. This systematic review of case reports/case-series showed that among patients treated with surgery and radiotherapy, 86.8% and 67.8% were disease free at follow-up. Surgery alone was associated with and OS of 80.3% at follow-up, while among the 80 patients treated with combined surgery and radiotherapy, the DFS was 66.7% [2].

The comparison of oncological outcomes between LVC and other laryngeal malignancies was available in a large cohort study [3]. Dubal et al. reported that the 1-, 5-, and 10-year DSS rates were 97.5%, 88.0%, and 77.4% for LVC, compared to 87.9%, 64.4%, and 50.4% for other laryngeal malignancies, respectively [3]. Authors showed that patients treated by surgery demonstrated significant higher 5-year DSS (90.2%) than patients who did not benefit from surgery (80.6%), regardless of radiotherapy use. The 5-year DSS of patients receiving primary radiotherapy and/or surgery were 92.3% for surgery alone, 85.6% for radiotherapy in combination with surgery, and 75.8% for radiotherapy alone [3].

### Bias analysis

Heterogeneity among studies in inclusion criteria, tobacco/alcohol use evaluations, comorbidities, LVC features and treatments are reported in Table 4. Most studies are retrospective studies (EL: IV). Only one study was prospective but uncontrolled [14]. Inclusion criteria were homogenous and reported in all studies. Authors included all cTNM stages [3, 8, 15–19, 21, 22, 24, 26] or only early stages in their studies [12, 14, 20, 23, 25]. The data about alcohol consumption and tobacco overuse were partly reported in 1 [23] and 5 studies [16, 19, 20, 23, 24], respectively (Table 4). Only Fliss et al. reported data about HPV infection through DNA analyses [17]. There was no full description of comorbidity features in studies. Few authors reported partial data of comorbidities, mostly for the oncological outcome interpretation [8, 12, 14, 20, 25, 26]. Clinical stages of LVC were fully reported in 13 studies according to anatomical location [3, 8, 15–17, 19, 21, 22, 24]. The details related to the surgical treatment, e.g. types of surgery and the realization of neck dissection, were available in 9 studies [8, 16, 17, 19], while the others reported partial information [3, 15, 21–23, 25]. Of the 12 studies investigating radiotherapy outcomes, the radiation protocols were fully or partly reported in 5 [8, 12, 17, 22, 24] and 3 studies [16, 21, 26], respectively. There was no information in 3 publications [3, 23, 25]. The oncological outcomes were reported considering the type of treatment in 11 studies [3, 8, 12, 14, 17, 18, 20, 23–26].

**Table 4:** Bias analysis

References	Tobacco	Alcohol	HPV	Como	Stage	Surgery	RT	Onco
Burns et al. [12]	No	No	No	Probably yes	Yes	Yes	Yes	Yes
Ferlito and Recher [26]	No	No	No	Probably yes	Probably yes	Yes	Probably no	Probably yes
Blakeslee et al. [14]	No	No	No	Probably no	Yes	Yes	–	Yes
Lundgren et al. [8]	No	No	No	Probably yes	Yes	Yes	Yes	Yes
Sllamniky et al. [15]	No	No	No	No	Yes	Probably yes	–	Yes
Hagen et al. [16]	Probably yes	No	No	No	Yes	Yes	Probably yes	Probably yes
Fliss et al. [17]	No	No	Yes	No	Yes	Yes	Yes	Yes
Longarela Herrero et al. [18]	No	No	No	No	Probably no	Yes	–	Yes
Maurizi et al. [19]	Probably no	No	No	No	Yes	Yes	No	Probably yes
Damm et al. [20]	Probably yes	No	No	Probably no	Yes	Yes	–	Yes
McCaffrey et al. [21]	No	No	No	No	Yes	Probably no	Probably yes	Probably yes
Strojan et al. [22]	No	No	No	No	Yes	Probably yes	Yes	Probably yes
Hod et al. [23]	Probably no	Probably no	No	No	Yes	Probably yes	No	Yes
Huang et al. [24]	Probably yes	No	No	No	Yes	–	Yes	Yes
Dubal et al. [3]	No	No	No	No	Yes	Probably yes	No	Yes
Jayakrishnan et al. [25]	No	No	No	Probably no	Probably yes	Probably yes	No	Yes

According to the bias tool used, the following points were considered: Tobacco/alcohol/HPV: yes = authors provided full details of tobacco or alcohol overuse or HPV infection; probably yes = authors provided partial details of all patients; probably no = authors provided partial details of some patients; no = no detail found. Comorbidities (como): yes = authors provided full comorbidity details of patient cohort; probably yes = authors provided selected comorbidity details of patients for the oncological outcome analysis; probably no = authors provided few details for few patients; no = no detail provided. Stage: yes = cTNM stages and anatomical locations of all tumors were provided; probably yes = authors provided incomplete cTNM stages or anatomical features of cohort; probably no = only cTNM or anatomical location were available for some patients; no cTNM/anatomical location details were provided. Surgery: yes = details of surgical treatments were reported (types of surgery for primary tumor AND neck dissection features); probably yes = some details of surgical treatment were provided for all patients (types of surgery OR neck dissection features); probably no = authors did not report the types of surgery or neck dissection features for all patients; no = no detail provided about the surgery or dissection features for any patient. Radiotherapy (RT): yes = full details of radiation treatment were provided for all patients (number of session AND doses AND type of RT); probably yes = partial details were found for all patients (number of session OR doses OR type of RT); probably no = partial details were provided for some patients (number of session OR doses OR type of RT); no = no detail of RT treatment. Oncological outcomes (onco): yes = oncological outcomes were reported for entire cohort and according to treatment; probably yes = oncological outcomes were provided for entire cohort without treatment detail; probably no = oncological outcomes were provided for some patients without treatment details; no = oncological outcomes were not reported

## Discussion

The number of publications dedicated to therapeutic and oncological outcomes of LVC did not increase over the past few decades with most of studies dating from the twentieth century. Yet, regarding the improvement of radiation protocols, it remains important for the future decades to better understand the differences between LVC and LSCC about surgery and radiotherapy-related oncological outcomes. In this systematic review, we investigated the epidemiological, pathological and oncological outcomes of LVC.

From an etiological standpoint, alcohol and tobacco overuses have long-time been recognized as important contributing factor of the LVC development. However, few studies reported full data information about tobacco and alcohol consumptions. Similar observation may be made for HPV detection in tumors of patients with LVC. Moreover, there was no large-cohort database study comparing the prevalence of alcohol, tobacco, and HPV outcomes between LVC and LSCC, which limits the improvement of etiological knowledge.

In this review, we reported that glottis is the main anatomical region of the LVC development, which may be an element supporting the better oncological outcomes of LVC compared to LSCC [3]. Indeed, glottic carcinomas are commonly diagnosed faster than supraglottic carcinomas according to the rapid impairments of voice quality [29]. From an oncological point of view, the glottic location may support the higher prevalence of early-stage tumors (cT1 and cT2) in LVC compared to LSCC patients [3] and the differences in survival outcomes. The investigations dedicated to the biology of LVC are rare [30, 31]. However, the biological differences between LVC and LSCC should be an additional element supporting the better prognosis of LVC compared to LSCC, as well as the low rate of distant metastasis. Precisely, we did not find report of distant metastasis in the systematic review of case-series, whereas only one study reported distant metastasis in LVC [32]. The occurrence of distant metastasis in this study should be treated with caution because the pathological diagnosis of LVC remains difficult with some LVC confounded with LSCC at the first pathological examination [32]. The lack of investigation of pathological biomarkers of LVC is an important issue for the diagnosis. Indeed, several studies supported that the pathological diagnosis remains difficult and may require several biopsies [8, 15, 22]. In the study of Stojan et al. the LVC diagnosis was carried out with only one biopsy for 36.6% of patients, whereas other patients needed repeated biopsies up to five times to have a definitive pathological diagnosis [22]. Similar findings were reported in the study of Lundgren et al. who made the diagnosis after one biopsy procedure in 21 patients (47.7%), while the 23 remaining individuals required a total of 68 endoscopic procedures to reach the definitive diagnosis [8]. The LVC may be particularly confounded with “hyperkeratosis”, which leads to delay in the diagnosis and the therapeutic management [15]. The challenging issue of histopathological diagnosis of LVC supports the risk of inclusion bias in many studies where some hyperkeratosis glottic (benign) lesions may have been considered as malignancies [15].

From a therapeutic standpoint, surgery has long time been considered as the main therapeutic option. The OS, DSS and RFS rates of studies included in the present review were better in surgery groups compared to radiation groups, which corroborated findings of previous systematic review [2] or large database studies [3, 25]. However, most case-series of patients treated with radiotherapy included data from 1970 to 2000. The radiotherapy techniques have considerably improved since the seventies, which led to higher OS, DSS, and RFS rates in patients treated after 2000 for laryngeal malignancies [33, 34]. Thus, the lowest effectiveness of radiotherapy in the management of LVC must be considered with prudence according to the lack of recent controlled study, the low number of patients treated with radiation in the past decade and the potential differences in patient profiles treated with radiotherapy *versus* surgery. In many studies, radiotherapy was proposed to patients with comorbidities or contraindication to surgery [8, 12, 19], which may bias the assessment of the radiation effectiveness. In the same vein, the post-radiation anaplastic transformation of LVC were reported in the seventies and eighties and concerned old radiotherapy approaches. Nowadays, it appears that the risk of post-radiation anaplastic transformation of LVC may be not considered as an important outcome for the treatment decisions. Because the growth of LVC is slow, it remains complicated to distinguish the evolution of an irradiated tumor versus recurrence [8]. All findings should be considered in future studies investigating the oncological outcomes of radiotherapy versus surgery. Moreover, treatments may differ from one center (country) to another; some physicians favoring surgery, while other prefer radiation when the situation allows several choices.

## Conclusion

LVC is a rare laryngeal cancer associated with better survival and recurrence outcomes than laryngeal squamous cell carcinoma. The superiority of surgery over radiotherapy is still not demonstrated regarding the lack of prospective controlled study using current radiation regimens.

## Abbreviations

DFS: Disease-free survival
DSS: Disease-specific survival
EL: Evidence-based level
LSCC: Laryngeal squamous cell carcinoma
LVC: Laryngeal verrucous carcinoma
OS: Overall survival
RFS: Recurrence-free survival
